# Sequence-Based Platforms for Discovering Biomarkers in Liquid Biopsy of Non-Small-Cell Lung Cancer

**DOI:** 10.3390/cancers15082275

**Published:** 2023-04-13

**Authors:** Liam J. Brockley, Vanessa G. P. Souza, Aisling Forder, Michelle E. Pewarchuk, Melis Erkan, Nikita Telkar, Katya Benard, Jessica Trejo, Matt D. Stewart, Greg L. Stewart, Patricia P. Reis, Wan L. Lam, Victor D. Martinez

**Affiliations:** 1 British Columbia Cancer Research Institute, Vancouver, BC V5Z 1L3, Canada; vg.souza@unesp.br (V.G.P.S.); aforder@bccrc.ca (A.F.); mpewarchuk@bccrc.ca (M.E.P.); ntelkar@bccrc.ca (N.T.); kbenard@bccrc.ca (K.B.); jtrejo@bccrc.ca (J.T.); mstewy@student.ubc.ca (M.D.S.); gstewart@bccrc.ca (G.L.S.); wanlam@bccrc.ca (W.L.L.); 2 Molecular Oncology Laboratory, Experimental Research Unit, School of Medicine, São Paulo State University (UNESP), Botucatu 18618-687, SP, Brazil; patricia.reis@unesp.br; 3 Department of Pathology and Laboratory Medicine, IWK Health Centre, Halifax, NS B3K 6R8, Canada; merkan@dal.ca; 4 Department of Pathology, Faculty of Medicine, Dalhousie University, Halifax, NS B3K 6R8, Canada; 5 Beatrice Hunter Cancer Research Institute, Halifax, NS B3H 4R2, Canada; 6 British Columbia Children’s Hospital Research Institute, Vancouver, BC V5Z 4H4, Canada; 7 Department of Surgery and Orthopedics, Faculty of Medicine, São Paulo State University (UNESP), Botucatu 18618-687, SP, Brazil

**Keywords:** bioinformatics, CTCs, ctDNA, high throughput, liquid biopsy, lung cancer, NGS

## Abstract

**Simple Summary:**

A lack of sensitive biomarkers hinders lung cancer detection and monitoring, resulting in late diagnosis and missed treatment response. Liquid biopsies have recently emerged as a promising method of detecting biomarkers in lung cancer patients without the need to conduct invasive procedures. New approaches for biomarker discovery have emerged due to advances in high-throughput sequencing technologies and bioinformatics tools. In this review, we comprehensively describe established and emerging methods for identifying lung cancer biomarkers using liquid biopsy. Furthermore, we highlight advanced bioinformatics tools and methods for processing NGS data, as well as recently developed software tailored for liquid biopsy biomarker detection.

**Abstract:**

Lung cancer detection and monitoring are hampered by a lack of sensitive biomarkers, which results in diagnosis at late stages and difficulty in tracking response to treatment. Recent developments have established liquid biopsies as promising non-invasive methods for detecting biomarkers in lung cancer patients. With concurrent advances in high-throughput sequencing technologies and bioinformatics tools, new approaches for biomarker discovery have emerged. In this article, we survey established and emerging biomarker discovery methods using nucleic acid materials derived from bodily fluids in the context of lung cancer. We introduce nucleic acid biomarkers extracted from liquid biopsies and outline biological sources and methods of isolation. We discuss next-generation sequencing (NGS) platforms commonly used to identify novel biomarkers and describe how these have been applied to liquid biopsy. We highlight emerging biomarker discovery methods, including applications of long-read sequencing, fragmentomics, whole-genome amplification methods for single-cell analysis, and whole-genome methylation assays. Finally, we discuss advanced bioinformatics tools, describing methods for processing NGS data, as well as recently developed software tailored for liquid biopsy biomarker detection, which holds promise for early diagnosis of lung cancer.

## 1. Introduction

Lung cancer is one of the most commonly diagnosed types of cancer and is the leading cause of cancer deaths worldwide [1]. Liquid biopsy, which involves analyzing body fluids to detect cancer-specific biomarkers, has emerged as a promising tool for the diagnosis and management of lung cancer patients [2,3,4,5]. One of the main advantages of a liquid biopsy test is that it provides a minimally invasive method for detecting and monitoring the disease. Traditional tissue biopsies are invasive, associated with pain and carry the risk of complications, such as infections. In contrast, a liquid biopsy test only requires the collection of body fluid, such as blood, which is a much less invasive procedure [2]. Liquid biopsy can be serially repeated, with additional advantages, such as speed, low cost and safety of isolating body fluids compared to tumor tissue [6]. Liquid biopsies are useful to detect cancer-specific DNA and other biomarkers that are shed into the bloodstream from cancer cells, providing a way to monitor the disease even in its early stages [7]. In fact, liquid biopsies are clinically applicable to guiding treatment decisions, such as determining the best therapeutic regimen or assessing if a patient’s cancer has changed or progressed [8]. Liquid biopsy tests are useful to monitoring response to treatment and detecting the emergence of drug-resistance mutations, allowing for treatment intervention and optimization. Liquid biopsy monitoring of drug response helps to improve overall outcomes and quality of life of lung cancer patients [2,9]. Therefore, liquid biopsy tests offer a number of important benefits for lung cancer patients, including improved accuracy and reliability of diagnosis, minimally invasive disease monitoring and the ability to guide and adjust treatment according to test results. The emergence of high-throughput sequencing technologies and bioinformatics tools has led to the development of new approaches for biomarker discovery using liquid biopsies [10,11,12]. High-throughput sequencing technologies allow for the simultaneous analysis of multiple biomarkers in a single sample, providing a comprehensive picture of the genomic landscape of a tumor. Bioinformatics tools applied to the analysis of large liquid biopsy datasets are key to identifying clinically actionable changes and predictive biomarkers to guide treatment decisions. Also, next-generation sequencing (NGS) technologies enable the simultaneous sequencing of millions of DNA fragments in a single test, providing a highly sensitive and specific method for detecting cancer-specific mutations, gene fusions and other genetic alterations associated with development and progression of lung cancer [13,14]. Such approaches are of utmost importance to improving the accuracy of lung cancer diagnosis and prognosis. The aim of this review is to survey the recent applications of NGS technologies to liquid biopsies in the context of non-small-cell lung cancer (NSCLC).

## 2. Sample Types and Materials for Biomarker Analysis

**Figure 1:**
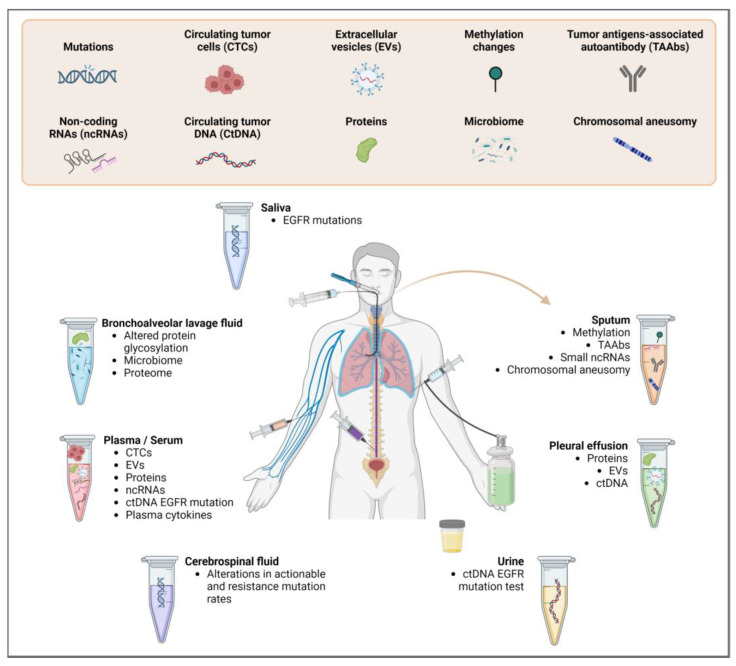
Types of biomarkers used to detect lung cancer in liquid biopsy and the types of bodily fluid samples from which they can be derived.

Liquid biopsy is a method that involves analyzing circulating tumor cells (CTCs) or molecules originating from the tumor in biological fluids [7]. These molecules include circulating tumor DNA (ctDNA) and RNA (ctRNA), proteins and extracellular vesicles (EVs) present in the biofluids of patients [7,15]. These biofluids include plasma, serum, sputum, bronchoalveolar lavage fluid (BAL), pleural effusion, urine, saliva and cerebral spinal fluid (CSF) [16] (Figure 1). Currently, the body fluid most commonly used for liquid biopsy in lung cancer is blood [16,17], which is used to identify specific biomarkers correlated with clinical outcomes in advanced NSCLC [18] and enables the monitoring of disease relapse and resistance of cancer [19]. Here, we briefly summarize materials used in liquid biopsy for biomarker analysis, as their features have been reviewed in depth elsewhere [7,15,16,20,21].

### 2.1. Circulating Tumor Cells (CTCs)

CTCs are isolated tumor cells or in clusters that are released by the primary tumor or metastatic cells that leak into the bloodstream towards the metastatic site [7]. CTCs are present only in extremely low concentrations in the blood, approximately one CTC per million leukocytes [22], but their presence can have diagnostic implications, for example, in lung cancer where high levels of CTCs have been associated with worse outcomes [23]. The presence of CTCs was also associated with low response rates, shorter progression-free survival and shorter overall survival in patients with advanced NSCLC treated with both targeted- and chemotherapy [24]. Different methods for enrichment, isolation and identification of CTCs have been developed according to the physical and biological characteristics of these cells [25,26,27]. In lung cancer, isolation by size of epithelial tumor cells (ISET) was the earliest size-based method used for CTC detection, showing high sensitivity and reproducibility [28]. Flow cytometry, fluorescence-activated cell sorting (FACS) and microfluidics are all commonly used techniques for isolating CTCs from liquid biopsy samples [29,30,31,32]. The most widely used immunomagnetic assay of epithelial cell adhesion molecule (EpCAM) enables the enumeration of CTCs of epithelial origin in whole blood, but it potentially misses a large cell population of “stem-like” characteristics [33,34]. Laser capture microdissection is another CTC sequestration method, which involves encapsulating a CTC on a hydrogel, extracting it with a laser and sequencing it [35].

### 2.2. Circulating Tumor DNA (ctDNA)

Circulating tumor DNA is part of the pool of total circulating cell-free DNA (cfDNA) and is released from tumor cells that have entered into apoptosis or necrosis [36]. In addition, fragments of tumor DNA resulting from the phagocytosis of necrotic tumor cells by macrophages can be released by these cells in circulation. The level of ctDNA present can vary by cancer type, but overall increases in ctDNA correspond with tumor burden, disease progression and metastasis [36,37,38,39]. Circulating tumor DNA is commonly quantified in serum and plasma samples, which are obtained by removing cellular components from whole blood samples by centrifugation, but it has also been reported in other bodily fluids, such as cerebrospinal fluid. The three main categories of methods to isolate the ctDNA, irrespective of the sample of origin, are phase isolation, silicon membrane-based spin column and magnetic bead-based isolation [36]. Once isolated, ctDNA can be analyzed by a variety of methods, for example, methylation markers can be measured using bisulfite sequencing [40]. Specific sequences and mutations can be targeted using polymerase chain reaction (PCR)-based methods, including conventional PCR and digital droplet PCR (ddPCR) [41,42]. Untargeted sequencing methods, such as shotgun massively parallel sequencing, can be used to quantify copy number alterations, and NGS can be used to screen for mutations in large regions of the genome [43,44]. It is important to note that standardization for pre-analytical factors, such as clotting, number of freeze-thaws, DNAse activity in the blood and the time elapsed between the blood draw and analysis is still required [7]. DNA input is also an issue, with low input also potentially impairing sensitivity [36].

### 2.3. Circulating Tumor RNA (ctRNA)

Circulating tumor RNA includes protein-coding RNAs (mRNAs), long non-coding RNAs (lncRNAs), microRNAs (miRNAs) and others. Circulating tumor RNA is most commonly collected from peripheral blood, but can also be detected in BAL fluid, saliva, pleural effusion, urine and CSF (largely in the form of miRNAs, the most abundant ctRNA) [15,45,46]. Both ctDNA and ctRNA contain mutational information, but ctRNA can also provide information about the quantitative expression levels of genes of interest [47]. Similar to ctDNA, ctRNA is likely released from apoptotic or necrotic cells but may also be released in exosomes (discussed below), which protect them from degradation [46,48]. RNAs that are not encapsulated are also released into bodily fluids, but certain classes (e.g., mRNAs and lncRNAs) are more likely to be degraded by ribonucleases (RNases) [15,49]. miRNAs, however, are less susceptible to this due to their small size and their association with RNA-binding proteins [15,50]. Circulating tumor RNAs can be isolated from liquid biopsies by using commercial RNA extraction kits, phenol-chloroform methods, or guanidium thiocyanate methods [51]. Kits may introduce biases, but this can be controlled by using exogenous RNA spike-ins [51]. After or prior to extraction, samples should also be treated with a DNase to prevent DNA contamination [51]. Following collection, ctRNAs can be analyzed using ddPCR, reverse-transcription quantitative PCR (RT-qPCR), microarray or NGS methods, such as RNAseq or small RNAseq [52,53]. For biomarker discovery, RNAseq/small RNAseq are the gold standard, while PCR-based methods are more commonly used for the detection of specific, predetermined ctRNAs [51,54].

### 2.4. Extracellular Vesicles (EVs)

EVs are small, membrane-bound vesicles that include both exosomes, which are secreted from the endosomal system of the cell, and microvesicles, which bud off the plasma membrane. They contain cargo, such as DNA fragments, non-coding RNAs, mRNAs and proteins that facilitate extracellular communication and thus tumor progression and metastasis through a variety of downstream mechanisms [55]. EVs are present at high levels in bodily fluids including blood and are stable in circulation, but isolating tumor-specific EVs is an ongoing challenge in the field [56]. Differential expression of surface markers on tumor-derived EVs, as well as differential loading of disease-related cargo into EVs, mean that EVs can be used as a tool for liquid biopsy for detection, monitoring of disease progression and prognosis [57,58,59]. Differential ultracentrifugation is currently the gold standard for EV isolation [60]. Newer approaches use immunoaffinity [61], and other physical properties or combinations of both [56]. Isolated EVs should be characterized with a Western blot for common EV-specific markers, such as CD63, CD81 and CD82 as well as at the single-exosome level with techniques, such as nanoparticle tracking or electron microscopy, to confirm their identity [62]. Isolated EVs can then be used for conventional protein analysis with Western blot, ELISA or novel methods, nucleic acid analysis with RT-qPCR or PCR-based technologies, such as digital droplet PCR and NGS [56].

## 3. Commonly Used Techniques for Biomarker Discovery in Lung Liquid Biopsy

There are several techniques that are commonly used for liquid biopsy, depending on the type of biomarkers being analyzed and the specific clinical application [15,63,64,65]. At present, ctDNA is the most extensively studied and widely used biomarker for liquid biopsy in NSCLC [66,67]. Both the European Medicines Agency (EMA) and the US Food and Drug Administration (FDA) have approved the use of ctDNA information for selecting NSCLC patients with EGFR-mutant cells for treatment with certain targeted therapies when a tumor sample cannot be evaluated [68,69,70]. Platforms and strategies currently available to detect ctDNA in lung cancer include both non–NGS- and NGS-based approaches [11,15,71].

### 3.1. PCR-Based Approaches

In general, the fraction of ctDNA in the blood of patients with cancer is thought to be relatively low, typically ranging from a few copies per milliliter to a few percent of the total DNA in the sample [72]. Consequently, traditional DNA analysis techniques (such as Sanger sequencing and pyrosequencing) are insufficient for detecting low amounts of DNA in blood samples [73]. PCR-based methods, such as real-time quantitative PCR (qPCR), digital PCR (dPCR) and mass-spectrometry-based methods, have been widely used as alternatives to traditional techniques since they have shown better sensitivities and are of low costs [2,11,15,74,75]. There are some variations of PCR-related methods including co-amplification at lower denaturation temperature-PCR (COLD-PCR) [76], refractory mutation system-PCR (ARMS-PCR) [77], locked nucleic acid (LNA)/DNA-PCR [78], peptide nucleic acid (PNA) clamp-PCR [79], beads, emulsions, amplification, magnetics (BEAMing) [80], ddPCR [81,82,83,84,85], intelligent multiplexed amplification for NGS applications (InPlex) [86,87] and Endpoint PCR [88]. The features of each method have been reviewed elsewhere [15,89]. Although the sensitivity of PCR-based methods is better when compared to traditional DNA analyses, it is still low, with the limit of detection of ctDNA ranging from < 0.0001% to 0.1% [15]. These techniques are also limited to the analysis of one or a very small number of genomic loci, even with multiplex analysis, and the specific mutation to be assayed is typically determined a priori [15,90,91]. While there have been promising uses of selective gene panels to screen ctDNA for oncogenic mutations in NSCLC patients, the sheer number of possible pathogenic mutations means that a predetermined panel is comparatively restricted in scope for biomarker discovery [92].

### 3.2. NGS-Based Approaches

To overcome the aforementioned limitations of PCR-based techniques, recent advances in NGS technologies have made it possible to detect even very low levels of ctDNA in the blood, allowing the detection of different types of alterations (e.g., point mutations, gene fusions and translocations) in multiple genes in the same analysis [11,39,93,94,95]. Currently, NGS technology has the ability to detect a minor allele frequency (MAF) of less than 1% in lung cancer [96]. Furthermore, the use of unique molecular identifiers (UMIs) or unique barcodes can help to improve the accuracy and sensitivity of NGS-based liquid biopsy assays for lung cancer, especially for samples with low ctDNA concentrations [97,98]. Targeted NGS approaches with molecular barcoding have been used to detect ctDNA in early-stage lung cancer patients. The assay was able to detect ctDNA in both patients with stage I or II NSCLC, though the sensitivity of the assay increased with higher ctDNA concentrations and was highest for patients with stage II disease [39]. Another study used a targeted NGS approach with UMIs to detect ctDNA in NSCLC patients. The assay was able to detect ctDNA in 59% of stage I or II NSCLC patients with a median MAF of 0.1% [98]. These studies demonstrated that the use of UMIs can reduce PCR and sequencing errors and improve the detection of low-frequency variants [98].

NGS uses probes to capture specific DNA fragments that are then sequenced in parallel and computationally aligned to a reference genome, enabling the sequencing of large numbers of gene targets and variant types in a single experiment. Furthermore, newer sequencing technologies provide options for sequencing portions of the genome at extremely high depths of coverage (i.e., the number of sequencing reads covering a specific position in the genome), which can reveal known mutations occurring at a low frequency or even uncover new driver mutations. Many methods are currently available to perform NGS analyses in liquid biopsy using either targeted or untargeted panels (Table 1). Targeted panels are designed to sequence specific genomic regions of interest, such as genes known to be frequently mutated in cancer. These panels can provide high coverage and depth of sequencing for the selected targets, which can increase the sensitivity of the assay for detecting low-frequency mutations. However, targeted panels may miss mutations outside of the selected regions, which can limit their ability to identify novel or unexpected mutations [8,11,15]. In contrast, untargeted panels, also known as whole-genome sequencing (WGS) or whole-exome sequencing (WES), can provide comprehensive and unbiased sequencing of the entire genome or exome, which can allow for the identification of novel or unexpected mutations [99]. However, untargeted panels may have lower coverage and depth of sequencing for specific regions, which can limit their sensitivity for detecting low-frequency mutations. Thus, both targeted and untargeted panels have advantages and limitations, and the choice of the panel may depend on the specific research or clinical question being addressed [8,11,15].

### 3.3. Clinically Validated Platforms for Biomarker Detection

In clinical practice, there are FDA-approved laboratory tests to analyze liquid biopsy biomarkers (typically ctDNA) for NSCLC. These include the Guardant360 CDx [100,101], FoundationOne Liquid CDx [102,103] and the cobas EGFR mutation Test v2 [104]. They use different panels to monitor a set of genes that may impact a patient’s response to drug treatments. The Guardant360 CDx test can identify the *EGFR* exon 20 insertion mutation and thus indicate if patients are eligible for treatment with amivantamab-vmjw [105,106]. The FoundationOne NGS test indicates whether gefitinib, osimertinib or erlotinib are the appropriate treatment, while the real-time PCR cobas EGFR mutation test v2 identifies specific *EGFR* mutations for erlotinib [102,103,104].

**Table 1:** NGS platforms designed for biomarkers in liquid biopsy.

Technology	Brief Description	References
**Non Targeted**		
WES	Whole Exome Sequencing sequences all exons in ctDNA for mutation detection. Less expensive than WGS (lower coverage). Sample requirement not always feasible in liquid biopsy.	[107,108,109]
Digital Karyotyping	Uses WGS to sequence short DNA tags and then aligns these tags to the reference genome to identify genomic alterations, e.g., CNVs, SNVs and SVs. The short DNA tags are typically generated by restriction enzyme digestion. Requires high-quality genomic DNA.	[43,110,111,112]
FAST-SeqS	Fast Aneuploidy Screening Test-sequencing System uses individual primer pairs to amplify the repeat regions of interest. The WGS version, called mFAST-SeqS, identifies any somatic mutations in the tumor and then uses those mutations as unique markers for monitoring the disease.	[113,114,115]
PARE	Personalized Analysis of Rearranged Ends uses WGS data to identify rearranged ends in ctDNA. Detects structural variations, e.g., translocations and inversions.	[111,116,117]
**Targeted panel**		
Tam-seq	Tagged-Amplicon deep sequencing uses primers targeting regions of interest for a pre-amplification step. Templates undergo individual amplification, aiding purification.	[44]
Safe-SeqS	Safe-Sequencing System is a method for profiling low-frequency mutations. The method combines PCR amplification of targeted genomic regions with UMIs and deep sequencing to achieve high accuracy and sensitivity. The use of UMIs reduces errors introduced by PCR amplification and sequencing.	[118]
CAPP-Seq	Cancer Personalized Profiling by Deep Sequencing uses a library that generates many hybrid affinities captures of recurrently mutated genomic regions to create the selector, which is used to identify individual-specific mutations in the tumor DNA. The selector is then applied to ctDNA for quantification.	[119]
Ion AmpliSeq™	Customized multiplex PCR amplifies target regions for analysis with the Ion Torrent sequencing platform.	[120]
Guardant360^®^	Analyzes 73 genes commonly mutated in cancer. Digital sequencing technology for mutation detection with 99.5% sensitivity and 99.999% specificity. FDA approval for use in patients with advanced cancer without treatment options.	[100,101]
Foundation One^®^CDx	Analyzes 324 genes and selects genomic signatures, including MSI and TMB. Detects single nucleotide variants, small in/dels, copy number alterations and gene fusions. FDA-approved for use in patients with solid tumors, including NSCLC, to sort patients for specific targeted therapies.	[102,103]
iDES	In Integrated Digital Error Suppression, DNA is tagged with UIDs and tracked through library preparation and sequencing for error correction. By incorporating UIDs into NGS, iDES can improve the accuracy and sensitivity of NGS assays, particularly in low-frequency variant detection.	[121]
TEC-Seq	Targeted Error Correction Sequencing is a method for profiling low-frequency mutations in cfDNA. Utilizes molecular barcoding to distinguish true mutations from false positive variants. Before any amplification, DNA fragments are tagged with different “exogenous” DNA barcodes. Additionally, the start and end genome mapping positions of paired-end sequenced fragments are used as “endogenous barcodes” to differentiate between individual molecules. This combination of barcodes enables tracking each fragment, allowing for the detection of rare mutations with high accuracy and sensitivity.	[98]

Abbreviations: CNVs: Copy Number Variations; ctDNA: circulating tumor DNA; FDA: US Food and Drug Administration; mFAST-SeqS: Mutation-focused Assessment of Sequencing and Tracking by Sequencing; MSI: microsatellite instability; SNVs: Single Nucleotide Variations; SVs: Structural Variations; TMB: Tumor Mutational Burden; UIDs: Unique Identifiers; UMIs: Unique Molecular Identifiers; WGS: Whole-Genome Sequencing.

## 4. Emerging Methods for Liquid Biopsy Biomarker Discovery

Emerging methods for biomarker discovery in liquid biopsy have the potential to revolutionize the ability to diagnose and monitor diseases non-invasively and to identify novel therapeutic targets. In this section, we describe some of the emerging methods for biomarker discovery in lung cancer liquid biopsy, including long-read sequencing, DNA methylation, single-cell sequencing and fragmentomics analysis.

### 4.1. Long-Read Sequencing

Current NGS platforms implemented in diagnostic settings (mainly developed by Illumina, San Diego, CA, USA) produce as many as 6 billion highly accurate (greater than 99.9%) “short reads” (150–400 nucleotides) per run [122]. Accuracy, parallelization and relatively low cost of short-read sequencing have made this method the preferred option for the detection of SNVs (single nucleotide variants) and small insertion–deletion mutations (indels) in liquid biopsies. The large number of generated reads allows for an improvement of the signal-to-noise ratio with regard to the detection of mutations in liquid biopsies. However, the clinical performance of short-read NGS is limited in the detection of any cancer-related feature associated with large fragments and/or duplicated regions of the genome. Newer long-read-based technologies such as Oxford Nanopore Technologies (ONT) (Oxford, UK) and PacBio (San Diego, CA, USA) have an advantage in detecting these features and avoiding amplification bias [123]. The most recent ONT devices use adaptors to attach phi29 DNA polymerase motor proteins to the ends of nucleic acid fragments, which feed the strands through ɑ-hemolysin and MspA pores. Bases are detected as they pass through the pores and change the flow of an electric current, which is output as sequencing data in real-time [124,125]. In PacBio Single Molecule Real-Time (SMRT) sequencing, each free DNA strand is circularized with adapters. A DNA polymerase replicates the strand with labeled nucleotides, releasing fluorescent signals that are read as a sequence in real-time [126,127]. Long-read sequencing enables the analysis of structural properties and the fragmentation patterns of cfDNA (including fragment size, nucleosome footprinting and methylation patterns), which can give information about their tissues of origin. This is known as “fragmentomics” [128,128,129,130], which we discuss further below. Long-read sequencing technologies (PacBio, ONT) were recently compared for real-time detection of long cfDNA in plasma. PacBio SMRT sequencing generated data with higher percentages of long cfDNA compared with nanopore sequencing. Yet a higher number of long cfDNA fragments eligible for the tissue-of-origin analysis could be obtained from ONT sequencing due to its much higher throughput [131]. Newer methods aim to obtain long-range information through alternative methods, such as linked-read sequencing (LR-seq). LR-seq is a cost-effective library preparation technology that maintains the long-range structure of the original genomic material in the generated short reads, making it an advantageous option that can be integrated into existing clinical lab protocols without additional specialized equipment [122,123,131,132,133].

### 4.2. DNA Methylation Markers

Profiling of DNA methylation (DNAm), the epigenetic mechanism by which a methyl group is transferred to a cytosine making a 5-methylcytosine, has proven to be highly informative in the detection and prediction of several disorders [134]. CpG dinucleotides (a cytosine followed by a guanine) are the most frequent cytosines where DNAm occurs, and methylation signatures observed in diseased tumor tissue show significant overlap in liquid biopsy samples (most often using ctDNA) [135,136,137]. On average, tumors show global hypomethylation when compared to healthy tissue, but loci-specific CpG island hypermethylation, along with enrichment in repressive histone marks, such as H3K27me3 [138,139].

Analysis of plasma ctDNA has been successful in detecting the aberrant hypermethylation of promoters in genes associated with the development of lung cancer, in single genes, and in gene signatures, detectable even at early cancer stages [140,141]. A recent study involving 2800 participants compared the accuracy and limit of detection of different ctDNA analysis techniques for early cancer detection and found that whole-genome methylation analysis strongly outperformed WGS and targeted sequencing [142]. Sputum samples have proven to be highly effective in detecting lung cancer progression early [143,144], specifically in active and former smokers. While relatively few studies have investigated DNAm changes occurring in lung cancer using urine, saliva and pleural effusion, they have still demonstrated the utility of using these liquid biopsy samples for early detection, as well as their success in separating lung cancer subtypes [145]. The majority of DNAm studies use bulk tissue; however, the methylomes of individual cell types differ, as demonstrated by cell-specific gene expression profiles [146]. Single-cell lung methylome profiling is still in its infancy [147].

DNAm profiling can be performed using sequencing-based or array-based methods. Whole genome bisulfite sequencing (WGBS), in which unmethylated cytosines are converted to uracil, remains the gold standard for DNAm detection. However, the method performs best for shorter-length fragments, is prone to errors due to bisulfite treatment and remains expensive [148]. Methylated DNA immunoprecipitation (MeDIP), which employs antibody ligation to methylated CpGs, works well for detecting genome-wide methylation enrichment and performs well for limited sample material [149]. Illumina’s Infinium HumanMethylation450 BeadChip array assessing 450,000 CpG sites and its successive Human MethylationEPIC BeadChip at 850,000 CpG sites currently are the most cost-effective and widely used.

### 4.3. Single-Cell Sequencing

Single-cell analysis has emerged as a powerful tool in liquid biopsy research, particularly for sequencing CTCs and cancer-associated immune cells [150,151,152,153,154]. Capturing and profiling a tumor cell in the act of metastasizing is a good strategy to understand the current molecular status of cancer [32]. There are several techniques for single-cell analysis in liquid biopsy, including single-cell RNA sequencing, single-cell DNA sequencing, single-cell proteomics, single-cell secretomes and single-cell metabolomics [151]. These methods can be used to identify heterogeneous subpopulations of cells, track their behavior and evolution and identify novel biomarkers [150,155].

Single-cell DNA sequencing can be used to detect genomic features, such as single nucleotide variants, copy number variation and microsatellite instability within CTCs. Single-cell RNA sequencing, which requires reverse transcription to cDNA, provides additional information on gene expression signatures. Following cell isolation, library construction is performed. During this process, whole-genome or whole-transcriptome amplification (WGA/WTA) is necessary to generate sufficient genetic material for sequencing, which requires numerous quality control measurements [151]. Some current WGA/WTA methods include multiple displacement amplification (MDA), multiple annealing and looping-based amplification cycles (MALBAC), emulsion whole-genome amplification (eWGA) and Laser-induced Isolation of Microstructure On transferrable-chip and sequencing (LIMO-seq) [156]. MALBAC offers higher uniformity of genomic amplification than MDA by utilizing quasilinear rather than nonlinear amplification. MALBAC has been successfully used for sequencing CTCs derived from NSCLC patients [157,158]. LIMO-seq involves using a microfluidic chip and a laser pulse to isolate CTCs, followed by MDA [159]. eWGA entails separating DNA fragments into droplets where separate MDA reactions occur [160]. A group has recently compared WGA and WTA methods for CTC single-cell sequencing and found that methods using MDA had higher amplification efficiency than others [161].

Following library construction, molecules are often labeled with a UMI. Libraries may then be pooled together (multiplexed) during sequencing, which necessitates demultiplexing based on barcodes and UMIs if present [162]. Multiplex PCR with predetermined gene panels has been used to profile CTC transcriptomes from lung cancer patients at the single-cell level, which revealed expression patterns correlated with prognosis [163,164]. While the low concentration of starting material in CTCs can pose a challenge to accurate sequencing, developments in WGA and WTA as well as the use of targeted panels have made it possible to capture the genetic diversity of metastatic tumor cells.

### 4.4. Fragmentomics

Fragmentomics in liquid biopsy has emerged as a promising approach for the detection and monitoring of cancer [165,166,167,168,169]. It involves analyzing the fragmentation patterns of cfDNA using NGS technologies (such as WES or WGS) without performing the DNA sonication step or using long-read sequencing [129,170,171]. Different computational and experimental approaches have been applied to study fragmentomics at varying resolutions and scales [169,170,171]. Various aspects of fragmentation can be taken advantage of, for example, length analysis and variant analysis. In cancer, it has been observed that ctDNA has a shorter median length and greater variability in size compared to cfDNA from healthy control subjects [171,172,173]. These findings suggest that fragmentomics analysis could be a useful tool for cancer diagnosis and monitoring, as the presence of ctDNA with abnormal size profiles may indicate the presence of cancer. Fragment length analysis of cell-free DNA can also be a useful tool for the detection of cancer-specific mutations. In a study of melanoma patients, the *BRAF V600E* mutation was found to occur more commonly at shorter fragment lengths (132–145 bp) than the wild-type allele (165 bp) [174]. In lung cancer patients, size-selecting for shorter cell-free DNA fragment lengths substantially increased the detection of the *EGFR T790M* mutant allele [174]. A study using WGS to analyze 344 plasma samples from 200 cancer patients showed that size-selected cfDNA identified clinically actionable mutations and copy number alterations that are otherwise not detected [175]. Additionally, the analysis of ctDNA fragments can be used to detect minimal residual disease (MRD) [176]. The combined analysis of both sequence variant and size fragmentation was reported to improve the stratification of patients into risk groups (low and high risk) for MRD in later-stage lung cancers (Stage II-IIIA) for guiding treatment decisions [177]. Fragmentomics data have also been used to detect early-stage lung cancer and to classify early-stage cancer patients [178,179,180].

## 5. Bioinformatics Pipelines for Analyzing Liquid Biopsy NGS Data

The process of generating and analyzing sequencing data using NGS technology involves three stages: primary, secondary and tertiary analysis [181,182]. The primary analysis involves the initial processing of raw data generated by the sequencing instrument, including base calling and quality control checks. The secondary analysis is focused on the pre-processed data, and it involves aligning the data to a reference genome and identifying genetic variants, such as single nucleotide polymorphisms (SNPs) or structural variations. In the tertiary analysis, the biological significance of the variants is interpreted and analyzed in the context of other available data sources [182].

### 5.1. Sequence Data Processing

During the primary analysis, the sequencing instrument generates a raw binary file containing the nucleotide bases identified during the sequencing run. For Illumina sequencers, the primary output is a BCL (Base Call) file, which contains the raw signal intensities for each base call of each read generated during the run. BCL files are then converted into a text-based (ASCII) FASTQ format file using tools such as BCLConvert [183]. The FASTQ file is the standard format for sequence data and is used for downstream analysis [184]. Several quality control metrics are evaluated to assess the quality of the sequencing run and the data generated [182,185,186,187]. The most common metrics include yield (the total number of reads generated in a run), quality scores (a measure of the confidence in the base call accuracy for each position in the sequence read) and error rates (calculated as the mismatches occurring during read alignment to the phiX spike-in).

In the secondary analysis, the pre-processed data generated during the primary analysis is aligned to a reference genome or assembled de novo [182,188,189]. This process can be computationally expensive and time-consuming due to the need to realign the 150-nucleotide-long reads to their original position in the human genome, which spans around 3 billion nucleotides. To address this challenge, various algorithms have been developed to effectively map the reads back to the human genome, such as BWA [190], Bowtie2 [191] and others. While these aligners can be used for both DNA and RNA data, the latter may require specific settings or have specializations depending on the type of data being aligned. For example, RNA-Seq data can be aligned using algorithms, such as Bowtie and BWA, but splice events result in specific requirements for transcriptome alignments, such as accounting for splice junctions and allowing for accurate mapping of reads to the appropriate isoform, which requires using tools such as TopHat2 [192], HISAT2 [193,194], HISAT-3N [195] or STAR [196]. The input for the secondary analysis generally involves FASTQ files generated during the primary analysis. The output file generated during the alignment process is a Binary Alignment Map (BAM) or a Sequence Alignment Map (SAM) file [197]. BAM files are currently the standard because their storage footprint is considerably reduced compared to SAM files.

### 5.2. Sequence Data Interpretation

#### 5.2.1. Biological Interpretation of the Sequencing Data

During the tertiary analysis stage, the processed data is annotated and interpreted, and several processes, such as variant calling, annotation and functional interpretation, take place to provide insights into the biological significance of the genetic variants identified [181,182]. Variant calling is the step where genetic variants, such as SNPs, indels and copy number variations (CNVs), are identified using tools such as GATK (Genome Analysis Toolkit), VarScan and SAMtools (Table 2) [198,199,200]. During the annotation process, publicly available databases, such as dbSNP, Ensembl, RefSeq, ClinVar, COSMIC and UCSC, are utilized to retrieve information regarding pathogenicity or significance from the identified genetic variants [201,202,203,204,205,206]. Tools, such as ANNOVAR, SnpEff and VEP (Variant Effect Predictor), are commonly used to position the genetic variants in a gene/transcript/genomic position context [207,208,209]. The functional interpretation step includes examining the functional relationships between genes and their involvement in biological pathways. Tools, such as IPA (Ingenuity Pathway Analysis), GSEA (Gene Set Enrichment Analysis), KEGG (Kyoto Encyclopedia of Genes and Genomes), Cytoscape, DAVID (Database for Annotation, Visualization and Integrated Discovery) and Enrichr are used to perform these tasks [210,211,212,213,214,215].

#### 5.2.2. Bioinformatic Platforms for Analyzing Long-Read Sequence Data

Bioinformatic analysis of long-read sequencing data from Oxford Nanopore Technologies (ONT) and PacBio is a rapidly evolving field, and neural nets are often integrated into the newest tools. Long-read data analysis has been thoroughly reviewed elsewhere [125,219,220], and a comprehensive searchable and filterable catalog of long-read data analysis tools has been created (https://long-read-tools.org, accessed on 1 March 2023). Some important steps in long-read sequencing analysis include base calling, quality control, error correction, genome alignment and detection of genetic alterations. Base calling for long-read sequencing is somewhat more error-prone than for short-read sequencing, but physical updates to the sequencers and error correction programs have improved accuracy. The main PacBio base calling program is called CCS [221], and the most commonly used ONT base calling program is Guppy though others such as Flappie, Scrappie and Taiyaki are sometimes used [222]. More specialized programs for data analysis can be found in the long-read tools catalog linked above.

#### 5.2.3. Analyzing DNA Whole-Genome Methylation Data

DNAm data numerically falls between 0 (no methylation) and 1 (complete methylation) per CpG, with intermediate methylation signifying CpGs with differential methylation. Methylation arrays (such as Illumina’s 450 k HumanMethylation450 updated to the 850 k HumanMethylationEPIC) measure the intensity of methylation via two types of probes: the original Infinium 1 (Type 1) probes, which utilize two beads per CpG to read the methylated and unmethylated intensity (red or green fluorescence per bead depending upon the methylation status), and the Infinium II (Type 2) probes which use one bead per CpG emitting either a green fluorescence for methylation or red fluorescence for no methylation. Based on the intensity of these red and green fluorescence levels, methylation is measured by either beta values (methylated divided by methylated + unmethylated + 100) or MValue (log of methylated divided by unmethylated) [223]. Processing steps involve validation of internal control probes for quality control metrics, prediction of sample sex by sex-specific probes on the X and Y chromosomes and normalization. Many software packages exist for DNAm analysis depending on the method of profiling, with most written in the R Language for Statistical Programming [224,225].

The biggest caveat of DNAm profiling is the low global coverage of genome-wide CpGs [226,227], wherein the majority of CpGs assessed are within gene bodies, CpG Islands, CpG shores and CpG shelves, leaving intergenic regions largely unprofiled. The accuracy of profiling methods also depends upon the CpG density of a genome [228]. Efficient and accurate DNAm results require rigorous quality control metrics during sample collection, sample processing and sample distribution while methylation profiling, as well as accounting for technical variation during analysis [229].

#### 5.2.4. Analyzing Single-Cell Sequence Data

Numerous platforms and software programs have been developed for processing single-cell sequencing data (Table 3), and a comprehensive searchable and filterable list is available online (https://www.scrna-tools.org/table, accessed on 1 March 2023). Frequently used platforms for quality control, visualization, and analysis of single-cell sequencing data include Seurat, Scanpy and Scater [230,231,232]. In most single-cell sequencing pipelines, to accomplish pre-processing and visualization, raw data are used to generate molecular barcodes for each cell. To remove reads from deceased and dying cells, quality control programs use molecular barcodes to measure covariates such as count depth, genes per barcode and mitochondrial genes per barcode, which can indicate unhealthy cells. Normalization is an important data processing step to account for artificial differences in expression observed due to sampling error during processing. Some normalization tools for single cells include MAST, SCDE and Basic [32]. The most frequently used formula for normalization is counts per million (CPM), which is the number of reads mapping to a particular feature divided by the total number of reads and multiplied by 10^6^. Transcripts per million (TPM) is another common normalization formula for Single-cell RNA sequencing (scRNAseq) data, which normalizes for both read depth and gene length. Visualization of the output is often improved by dimensionality reduction methods, such as t-distributed stochastic neighbor embedding (t-SNE), which reduces the noise of differences between cells and better resolves similarities, as well as Uniform Manifold Approximation and Projection (UMAP) [233]. After these processing steps, downstream analysis is possible. This tends to focus on clustering cells by shared traits or cell trajectory inference, which entails mapping cell state changes over time. Clustering is often carried out using a k-means clustering algorithm. The popular Seurat platform uses the Louvain algorithm with K-nearest-neighbor graphs [162]. Annotation of the identified cell clusters can be aided with databases such as The Human Cell Atlas [234], CellMarker [235] and automated cluster annotation platforms, such as scmap, can be used [236]. Cell trajectory inference can be carried out with Monocle, Wanderlust or Slingshot [237,238,239]. For differential gene expression analysis between clusters, tools such as DESeq2 or EdgeR may be used [240,241].

#### 5.2.5. Other Software for Analyzing Liquid Biopsy Samples

Some tools have been developed specifically for long-read cfDNA sequencing data from ONT devices. A program called cfNano uses deconvolution to detect cancer-associated signatures in ctDNA, including nucleosome footprinting, copy number alterations, methylation changes and fragmentation patterns [250,251]. Another program called FrEIA uses variations in the sequences at the ends of cfDNA fragments to improve the sensitivity of cancer signal detection [252,253]. Nanomonsv is designed to detect somatic cancer-associated structural variants in paired tumor and normal samples [254,255]. Nanovar is a SV caller with the ability of detecting variants from low-depth, long-read sequencing (homozygous SVs can be detected using 4×, while heterozygous SVs are detected at a threshold of 8×) [256].

The development of bioinformatic tools to sequence single CTCs often includes quality control to account for errors introduced during whole genome or whole exome amplification. Several tools exist for different types of CTC studies [32]. Monovar is a variant caller for single-cell data [257]. OncoNEM and SCITE are designed to use single-cell data to trace the evolutionary trajectory of cancers [258,259]. RaceID2 and GiniClust are tools for identifying a cell’s tissue of origin based on single-cell sequencing [260,261]. Ginkgo is a web platform that is used to identify copy number variants in single-cell sequencing data to construct a phylogenetic tree [262], which has been used to study the heterogeneity of CTCs [263].

cfDNApipe is an integrated tool for WGS or bisulphite cfDNA sequencing data that performs quality control and finds differentially methylated regions, copy number variation and alterations in fragment lengths, which facilitates tissue of origin analysis or fragmentomic analysis [264,265].

## 6. Conclusions

The use of liquid biopsy and NGS technology has revolutionized the diagnosis and management of lung cancer, providing a non-invasive alternative to traditional solid tissue biopsies. Liquid biopsy enables the detection of cancer-specific mutations and other biomarkers in a minimally invasive manner, allowing for serial testing and monitoring of lung cancer progression or response to treatment.

While the use of liquid biopsy and NGS technology has brought numerous benefits to the field of lung cancer diagnosis and management, there are also some limitations and challenges. One of the major limitations compared to solid tissue biopsies is the signal-to-noise ratio. The low abundance of circulating tumor DNA/RNA, circulating tumor cells and extracellular vesicles in biological fluids, detecting cancer-specific mutations and other biomarkers, can be problematic, as these signals can easily be drowned out by processing protocols, background noise and/or sequencing errors. As a result, the sensitivity and specificity of liquid biopsy and NGS-based analysis can be affected, leading to false-positive or false-negative results. Consequently, one of the main challenges is the need for increased sensitivity and specificity of liquid biopsy analysis, particularly in detecting early-stage lung cancer or minimal residual disease. This is especially important for patients who are not eligible for surgery or who have a high risk of recurrence.

NGS technology has enabled a comprehensive and highly detailed analysis of liquid biopsy samples, providing valuable information for cancer diagnosis, monitoring and treatment selection. With its ability to analyze multiple types of genetic alterations (including SNVs, indels and copy number/structural variations) and biomarkers simultaneously, NGS has opened up new avenues for identifying potential therapeutic targets and developing precision medicine strategies. Moreover, emerging sequencing technologies, such as long reads sequencing and single-cell sequencing, are expected to expand the nature of biomarkers that can be detected in liquid biopsy. Long reads sequencing has the potential to detect complex genomic rearrangements at an unprecedented level of detail, while single-cell sequencing has the potential to provide a more comprehensive analysis of the heterogeneity of cancer cells in liquid biopsy, which can play a significant role in the development of resistance to treatment. The combination of current NGS approaches and emerging sequencing technologies is expected to further enhance the precision and efficacy of lung cancer diagnosis and treatment.

In conclusion, the use of NGS in the analysis of liquid biopsy has revolutionized lung cancer diagnostics and management; however, limitations and challenges remain to fully realize its potential. Further research will improve the ability of NGS to further unlock the biological information contained in liquid biopsies.

## Figures and Tables

**Table 2:** Tools for computational analysis of NGS data.

Tool	Description	Resource	References
**Variant calling**			
GATK	Genome Analysis Toolkit has multiple applications, e.g., variant discovery, genotyping and mutation detection, quality control, coverage analysis and error correction.	https://gatk.broadinstitute.org/hc/en-us (accessed on 1 March 2023)	[198]
VarScan	Detects and characterizes variants, e.g., SNPs, indels and somatic mutations in tumor-normal pairs. Identifies low-frequency variants using Bayesian algorithms and statistical models for sensitivity and specificity.	https://varscan.sourceforge.net/ (accessed on 1 March 2023)	[199]
Vardict	Detects SNVs, indels and CNVs from tumors and tumor-normal pairs. Uses a combination of local realignment, base quality score recalibration and variant calling algorithms to identify variants. Handles data with high variability, e.g., low-coverage or high tumor heterogeneity.	https://github.com/AstraZeneca-NGS/VarDict (accessed on 1 March 2023)	[216]
Samtools	Analyzes alignment files in multiple formats, e.g., BAM, SAM and CRAM. Performs file conversion, sorting, indexing, filtering and merging. Quality control, coverage analysis and variant calling for reference genomes and alignment algorithms.	https://www.htslib.org/ (accessed on 1 March 2023)	[200]
Strelka2	Heuristic approach to detect SNVs, indels and structural variants. Employs a combination of probabilistic and machine learning methods to detect somatic mutations while minimizing false positives. Uses local assembly-based variant calling to improve variant detection sensitivity in regions with low read coverage or high levels of noise.	https://github.com/Illumina/strelka (accessed on 1 March 2023)	[217]
ANNOVAR	Enables genetic variants annotation in various genome builds, e.g., RefSeq, dbNSFP and gnomAD. Allows filtering and prioritization of variants based on the functional impact, population frequency, etc.	https://annovar.openbioinformatics.org/en/latest/ (accessed on 1 March 2023)	[207]
**Variant annotation**			
SnpEff	Annotation and functional analysis of genetic variants. Predict the effects of genetic variants on genes, transcripts and regulatory regions and classify variants based on their impact.	http://pcingola.github.io/SnpEff/ (accessed on 1 March 2023)	[208]
VEP	Variant Effect Predictor performs analysis, annotation and prioritization of genomic variants in coding and non-coding regions	https://useast.ensembl.org/info/docs/tools/vep/index.html (accessed on 1 March 2023)	[209]
**Functional interpretation**			
GSEA	Gene Set Enrichment Analysis identifies enriched biological pathways, functions and processes based on the expression profiles of genes in a sample or dataset.	https://www.gsea-msigdb.org/gsea/index.jsp (accessed on 1 March 2023)	[210]
KEGG	Kyoto Encyclopedia of Genes and Genomes is a data and knowledge base of biological systems, e.g., metabolic pathways, regulatory networks and genetic information. A comprehensive set of reference genomes, gene annotations and pathway maps.	https://www.genome.jp/kegg/ (accessed on 1 March 2023)	[211]
Cytoscape	Open-source software for the visualization, analysis and interpretation of complex biological networks. Utilizes various data types, e.g., genetic, genomic, proteomic and metabolomic.	https://cytoscape.org/ (accessed on 1 March 2023)	[212]
DAVID	Resource for functional annotation and analysis of biological data. A comprehensive set of functional annotation tools, including gene ontology/pathway analysis and functional annotation clustering.	https://david.ncifcrf.gov/ (accessed on 1 March 2023)	[213]
Enrichr	Web-based analysis tool. Provides visualization summaries of collective functions of gene lists. Integrates public databases and annotations for identification and annotation of biological pathways, functions and processes associated with a set of genes or proteins.	https://maayanlab.cloud/Enrichr/ (accessed on 1 March 2023)	[214]
GeneMania	Identifies and analyzes functional gene networks. Uses combinations of functional genomics data sources, including protein-protein interactions, co-expression, genetic interactions and pathways to construct gene networks related to the biological function or disease.	http://genemania.org/ (accessed on 1 March 2023)	[215,218]
IPA	Ingenuity Pathway Analysis identifies key biological pathways, networks and functions associated with gene or protein sets. A range of visualization and reporting features. Supports various input and output file formats.	https://digitalinsights.qiagen.com/products-overview/discovery-insights-portfolio/analysis-and-visualization/qiagen-ipa/ (accessed on 1 March 2023)	Not applicable

Abbreviations: BAM: Binary Alignment Map; CNVs: Copy Number Variants; CRAM: Compressed Reference-oriented Alignment Map; indels: Insertion–deletion mutations; SAM: Sequence Alignment Map; SNPs: Single-nucleotide polymorphisms.

**Table 3:** Platforms designed for bioinformatic analysis of single-cell sequencing data.

Tool	Brief Description	Resource	References
Seurat	R-based platform for raw data processing, paired sample analysis and visualizations. Uses machine learning and clustering algorithms to identify biological features. Assesses cellular heterogeneity via normalization, dimensionality reduction and integration tools.	http://satijalab.org/ (accessed on 1 March 2023)	[230,242]
Monocle	R-based scRNA-Seq analysis software. It uses algorithms and machine learning to determine cell developmental trajectories, identify molecular pathways and track changes in gene expression.	http://cole-trapnell-lab.github.io/monocle-release/ (accessed on 1 March 2023)	[237,243,244]
ChromVAR	R package for analyzing variations in chromatin accessibility in scATAC-Seq data to identify associated motifs or genomic annotations. It uses visualization techniques to detect and highlight changes in gene expression and provides users with powerful statistical methods. It is also capable of detecting and correlating molecular pathways.	https://greenleaflab.github.io/chromVAR/ (accessed on 1 March 2023)	[245]
DRAGEN Single-Cell RNA Pipeline	Cloud-based platform to analyze scRNA-Seq data: aligning and mapping reads, detecting features and biomarkers and generating visualizations. It processes multiplexed scRNA-Seq datasets from reads to a cell-by-gene UMI count gene expression matrix. Features splice-aware RNAseq alignment and matching to annotated genes for transcript reads, cell-barcode and UMI error correction and QC metrics.	http://illumina.com/ (accessed on 1 March 2023)	Not applicable
Tapestri	Pipeline to analyze scRNA-Seq data generated by the Tapestri platform. It Includes sequence import, data analysis and visualization capabilities. The software enables variant identification, including SNVs and CNVs, at clonal and subclonal levels.	https://support.missionbio.com/hc/en-us/categories/360002505454-Tapestri-Insights (accessed on 1 March 2023)	Not applicable
Scanorama	Integrates data from heterogenous scRNA-seq experiments via detecting common cell types among datasets. Identifies datasets, e.g., cells with similar transcriptional profiles, and leverages the matches for batch correction and integration. Can handle different dataset sizes and sources and does not require all datasets to share a cell population.	https://cb.csail.mit.edu/cb/scanorama/ (accessed on 1 March 2023)	[246]
scmap v1.1.5	An R package that projects cells from a scRNA-Seq data set onto cell types or individual cells from various experiments. It is a widely applicable projection method, detecting the best-matching cell type or individual cell in the reference. It allows fast feature selection, centroid calculation and index creation.	https://scmap.sanger.ac.uk/scmap/ (accessed on 1 March 2023)	[236]
Scrublet v0.1	Single-Cell Remover of Doublets, acronym Scrublet, is a framework for predicting the effect of multiplets in analysis and also identifies problematic multiplets. It can identify neotypic multiplets for an analyzed dataset. The Scrublet classifier can implement arbitrary functions for preprocessing and embedding of single-cell data.	https://github.com/AllonKleinLab/scrublet (accessed on 1 March 2023)	[247]
CellRanger v2.2.0	A set of analysis pipelines that can process Chromium single-cell data to align reads, generate feature-barcode matrices, perform clustering, amongst other tasks. It contains five pipelines for the 3′ Single Cell Gene Expression Solutions and similar products.	https://support.10xgenomics.com/single-cell-gene-expression/software (accessed on 1 March 2023)	Not applicable
CITE-seq-count v1.2	Python package that aids counting antibody tags from CITE-Seq or cell hashing experiments.	https://github.com/Hoohm/CITE-seq-Count (accessed on 1 March 2023)	[248]
Drop-seq tools v2.0.0	Java tool to analyze profiling of individual cells (UMI cells encapsulated in droplets for oligodT sequencing). Libraries produce paired-end reads (read 1: cell barcode and UMI; read 2: cDNA sequence) for identity and abundance of the transcripts in each cell.	https://github.com/broadinstitute/Drop-seq (accessed on 1 March 2023)	Not applicable
deepTools v3.1.2	Galaxy-based web server for processing and visualizing deeply sequenced data. Allows completion of bioinformatic workflows to integrative analyses. Supports four tasks: quality control, data processing and normalization, data integration and visualization.	Not applicable	[249]

Abbreviations: CITE-Seq: Cellular Indexing of Transcriptomes and Epitopes by Sequencing; CNV: Copy number variant; OligodT: Oligo-deoxythymidine; QC: Quality control; scATAC-Seq: Single-cell Assay for Transposase-Accessible Chromatin using sequencing; scRNA-Seq: Single-cell ribonucleic acid sequencing; SNV: Single nucleotide variant; UMI: Unique molecular identifier.

## Data Availability

Not applicable.
